# The role of moderate-to-vigorous physical activity in mediating the relationship between central adiposity and immunometabolic profile in postmenopausal women

**DOI:** 10.1590/2359-3997000000259

**Published:** 2017-03-20

**Authors:** Tiego A. Diniz, Fabricio E. Rossi, Loreana S. Silveira, Lucas Melo Neves, Ana Claudia de Souza Fortaleza, Diego G. D. Christofaro, Fabio S. Lira, Ismael F. Freitas

**Affiliations:** 1 Universidade de São Paulo São Paulo SP Brasil Universidade de São Paulo (USP), São Paulo, SP, Brasil; 2 Departamento de Educação Física Universidade Estadual Paulista Presidente Prudente SP Brasil Grupo de Imunometabolismo e Exercício, Departamento de Educação Física, Universidade Estadual Paulista (Unesp), Presidente Prudente, SP, Brasil; 3 Centro de Estudos e Laboratório de Avaliação e Prescrição de Atividades Motoras Departamento de Educação Física Unesp Presidente Prudente SP Brasil Centro de Estudos e Laboratório de Avaliação e Prescrição de Atividades Motoras (Celapam), Departamento de Educação Física, Unesp, Presidente Prudente, SP, Brasil; 4 Departamento de Educação Física Unesp Presidente Prudente SP Brasil Grupo de Estudos em Atividade Física e Saúde, Departamento de Educação Física, Unesp, Presidente Prudente, SP, Brasil

**Keywords:** Postmenopause, insulin resistance, cytokines, adiponectin, adipose tissue, obesity

## Abstract

**Objectives:**

To analyze the role of moderate-to-vigorous physical activity (MVPA) in mediating the relationship between central adiposity and immune and metabolic profile in postmenopausal women.

**Materials and methods:**

Cross-sectional study comprising 49 postmenopausal women (aged 59.26 ± 8.32 years) without regular physical exercise practice. Body composition was measured by dual-energy X-ray absorptiometry. Fasting blood samples were collected for assessment of nonesterified fatty acids, tumor necrosis factor-α (TNF-α), interleukin-6 (IL-6), adiponectin, insulin and estimation of insulin resistance (HOMA-IR). Physical activity level was assessed with an accelerometer (Actigraph GTX3x) and reported as a percentage of time spent in sedentary behavior and MVPA. All analyses were performed using the software SPSS 17.0, with a significance level set at 5%.

**Results:**

Sedentary women had a positive relationship between trunk fat and IL-6 (rho = 0.471; p = 0.020), and trunk fat and HOMA-IR (rho = 0.418; p = 0.042). Adiponectin and fat mass (%) were only positively correlated in physically active women (rho = 0.441; p = 0.027). Physically active women with normal trunk fat values presented a 14.7% lower chance of having increased HOMA-IR levels (β [95%CI] = 0.147 [0.027; 0.811]).

**Conclusions:**

The practice of sufficient levels of MVPA was a protective factor against immunometabolic disorders in postmenopausal women.

## INTRODUCTION

Menopause contributes to changes in body composition, particularly increased central adiposity ( 1 ). It is also associated with decreased physical activity levels ( 2 ), which can favor the development of obesity ( 3 ). In contrast, engaging in recommended (≥ 150 minutes per week) moderate-to-vigorous physical activity (MVPA) ( 4 ) has been associated with improved body composition profile, including decreased body fat and increased lean mass ( 5 , 6 ).

Obesity is currently considered a public health problem. It is responsible for the development of chronic low-grade inflammation ( 7 ), a condition that is characterized by increased plasma endotoxin ( *i.e.* , lipopolysaccharide), saturated fatty acids, and proinflammatory factors, which are involved in the development of morbidities such as type 2 diabetes mellitus, hypertension, dyslipidemia and metabolic syndrome, and decreased anti-inflammatory mediators ( 8 , 9 ). Taken together, these comorbidities may increase the likelihood of cardiovascular diseases, one of the leading causes of death worldwide ( 10 ).

Insufficient levels of physical activity also increase plasma concentrations of inflammatory mediators such as interleukin (IL)-6 and tumor necrosis factor (TNF)-α, and decrease adiponectin levels and insulin sensitivity ( 11 , 12 ). We have previously shown that postmenopausal women with two or more risk factors, including low MVPA levels, are more likely to have metabolic abnormalities such as insulin resistance ( 13 ). As aforementioned, central adiposity is one of the main factors responsible for increased proinflammatory mediators, displaying a linear relationship with these mediators. However, little is known about the protective role of MVPA in regards to the association of central adiposity, proinflammatory mediators, and metabolic profile in postmenopausal women.

Thus, the purpose of the present study was to analyze the role of MVPA in mediating the association between central adiposity and the immune and metabolic profile of postmenopausal women.

## MATERIALS AND METHODS

### Sample

This was a cross-sectional study conducted between 2013–2014 in a city with ~220,000 inhabitants and a human development index of 0.846, located in the west part of the state of São Paulo, southeastern Brazil ( 14 ).

To be included in the present study, the participants had to meet the following criteria: i) be postmenopausal (absence of a menstrual cycle for at least 1 year and follicle-stimulating hormone [FSH] > 30 IU/L) ( 15 ); ii) be older than 50 years on the date of the assessment; iii) have not engaged in regular physical exercise ( *e.g* ., walking/running, strength training, etc.) for at least 6 months prior to the study; iv) not be receiving hormone replacement treatment; v) not be using drugs such as beta-blockers, statins, etc.; vi) have signed the written informed consent form for study participation.

We performed a power analysis for the design of this study, based on the observation from a previous study that verified a correlation between time of physical activity practice after spinal cord injury and HOMA-IR (r = -0.591; p = 0.033) ( 16 ). Using a power of 0.80% and a type I error of 0.05, according to Miot ( 17 ), the required sample size was estimated at 21 subjects; however, we opted for over-recruitment.

A total of 77 women were evaluated, but only 60 met the study inclusion criteria. After assessing the physical activity levels of the participants using accelerometry, we found that 11 of them had not used the device for the prescribed minimum number of days (4 days during the week and 1 day during the weekend). Thus, the final sample selected for the analysis comprised 49 women.

All procedures used in this study met the criteria of the 196/96 Resolution on Ethics in Research of the Brazilian National Health Council (Brasília, DF). All participants included in the study signed an informed consent form approved by the Research Ethics Committee at the university linked to the project (Protocol: 64/2011).

### Data collection

#### Anthropometry and body composition

During anthropometric measurement, all participants wore light clothing and remained barefoot. The height of the subjects was measured using a fixed stadiometer (Sanny, São Bernardo do Campo, São Paulo, Brazil) with an accuracy of 0.1 cm. Body weight was measured using a digital scale (Filizola PL 50, Filizola Ltda., Brazil) with an accuracy of 0.1 kg.

Body fat was analyzed with dual-energy X-ray absorptiometry (DXA; Lunar DPX-NT, version 4.7, General Electric Healthcare, Little Chalfont, Buckinghamshire, United Kingdom). Each examination lasted for approximately 15 minutes. During the examination, the participants were positioned in the supine position on the scanner. The values were expressed as a percentage of body fat (%BF), and trunk and abdominal fat, all in kilograms.

#### Non-exercise physical activity

The habitual levels of physical activity of the participants were assessed using a triaxial accelerometer sensor (Actigraph model GT3X, Actigraph LLC, Pensacola, Florida, United States), which recorded the movements in the three orthogonal planes: vertical, horizontal anteroposterior, and horizontal mediolateral. To carry out the measurements, the accelerometers were attached to an elastic tape and placed on the subjects’ waist, above the hip, at the height of the iliac crest on the right side of the body. The participants were required to use the accelerometer for 7 days, and received instructions for using it during all waking hours, except during bathing or aquatic activities such as swimming ( 5 ).

A specific software (ActiLife5, data analysis software by Actigraph) was used to process the obtained data, and only results obtained during full monitoring days were analyzed. A non-wear time was defined as at least 60 consecutive minutes with zero counts, with an allowance of up to 2 minutes of counts between 0 and 100 ( 18 ). A valid day was defined as ≥ 10 hours of monitor wear time, and only participants with ≥ 5 valid days (including at least 1 day during the weekend) were included in the present analyses ( 19 ).

Raw measurements from the accelerometer were determined as *counts* , which was an arbitrary measurement: the greater the number of counts, the higher the level of physical activity. *Counts* from each sample were added over a specific period of 60 seconds, called an *epoch* . The period of 60 seconds was chosen for this study population due to the type of physical activity, which is characterized by a low intensity and long duration pattern ( 20 ).

In an attempt to obtain a biological value and facilitate the interpretation of data (counts per minute [cpm]) provided by the accelerometer, cpms were translated into physical activity minutes. To classify the intensity of the physical activity, we used the recommendations for accelerometers proposed by Freedson and cols *.* ( 21 ). We defined the physical activities as moderate for values between 1952–5724 cpm (from 3.00 to 5.99 METs), as vigorous for values between 5725–9498 cpm (6.00 to 8.99 METs), and as very vigorous for values above 9499 cpm (≥ 9 METs). We also used Freedson cut points to categorize an epoch as sedentary (< 100 cpm).

Thereby, the practice of habitual physical activity was expressed in percentage of time in sedentary behavior and MVPA. In addition, women who engaged in less than 150 minutes of MVPA were considered to be sedentary, according to recommendations by the American College of Sports Medicine (ACSM) ( 4 ).

## Blood collection and inflammatory and metabolic profile

After a 12-hour fast, blood samples were collected by nurses in sterile tubes containing heparin, an anticoagulant for blood samples. Triacylglycerol (mg/dL) and FSH levels were processed in a private laboratory in the city where the study was conducted. Nonesterified fatty acids were assessed by a colorimetric method with a commercial kit (ZenBio Inc., Research Triangle Park, North Carolina, United States). Serum insulin, IL-6, TNF-α, and adiponectin were quantified using enzyme-linked immunosorbent assay (ELISA) with a commercial kit (RayBio^**®**^ Human ELISA Kit, Norcross, Georgia, United States), as per the manufacturer’s manual. In addition, the HOMA-IR and HOMA-β were calculated using a previously reported method ( 22 ).

## Statistical analysis

The Kolmogorov-Smirnov test was used to test the normality of the data. Trunk fat groups were compared by independent Student’s *t* -test and corrected with Levene’s test for equality of variance. The proportion of women meeting the ACSM physical activity recommendation (MVPA ≥ 150 min/week) in each trunk fat group was evaluated with McNemar’s test. Spearman’s correlation (rho) was used to analyze the relationship between levels of physical activity and markers of systemic inflammation. In addition, odds ratios were obtained using binary logistic regression to analyze the magnitude of possible associations between MVPA practice, inflammation markers, and trunk fat.

All analyses were performed using the statistical software SPSS (version 17.0). The level of significance was set at 5%.

## RESULTS

Mean FSH levels were 59.33 **±** 23.89 mUI/mL. Table 1 shows the baseline characteristics of the sample dichotomized by central adiposity. As expected, women with increased trunk fat had increased amount of total body fat and higher insulin resistance when compared with those with normal trunk fat. Interestingly, there was no difference between time in sedentary behavior in the trunk fat groups; however, participants in the normal trunk fat group accumulated approximately 20 minutes more than the other group, although the difference did not reach statistical significance. In addition, of a total of 49 postmenopausal women, 25 (51%) were physically active and 24 (49%) were sedentary according to the ACSM.

**Table 1:** Baseline characteristics of the sample dichotomized by central adiposity

Variable	Normal trunk fat (kg) (Lower median)	Increased trunk fat (kg) (Upper median)	p-value
Age (years)	59.80 (6.07)	59.07 (5.24)	0.652
FSH (mUI/mL)	61.48 (19.16)	57.38 (27.82)	0.594
Weight (kg)	60.31 (5.72)	79.66 (8.54)	**< 0.001**
Height (cm)	154.75 (5.15)	157.70 (6.02)	0.073
BMI (kg.m^-2^)	25.20 (2.29)	32.07 (3.40)	**< 0.001**
Body fat (%)	40.16 (4.69)	48.32 (4.31)	**< 0.001**
Trunk fat (kg)	11.88 (2.97)	20.68 (2.88)	**< 0.001**
Abdominal fat (kg)	2.05 (0.86)	3.50 (0.61)	**< 0.001**
NEFA (µM)	192.45 (48.58)	199.64 (59.47)	0.646
TNF-α (pg/mL)	288.51 (108.86)	234.87 (141.31)	0.143
IL-6 (pg/mL)	105.50 (11.70)	108.12 (8.65)	0.375
Fasting plasma insulin (μIU/ml)	11.81 (9.85)	16.51 (13.92)	0.181
HOMA-IR	2.47 (1.94)	4.57 (3.93)	**0.022**
Adiponectin (μg/mL)	0.24 (0.17)	0.30 (0.35)	0.492
Sedentary behavior (hours/week)	118.45 (12.92)	117.78 (15.92)	0.871
MVPA (min/week)	190.62 (133.39)	171.97 (114.28)	0.601
MVPA ≥ 150 min/week (%)	54.2%	48.0%	0.500†

FSH: follicle stimulating hormone; BMI: body mass index; NEFA: nonesterified fatty acids; TNF-α: tumor necrosis factor-α alpha; IL-6: interleukin-6; HOMA-IR: homeostatic model assessment-insulin resistance; HOMA-β: homeostatic model assessment-β-cell function; MVPA: moderate-to-vigorous physical activity. † McNemar’s test.

**Figure 1:**
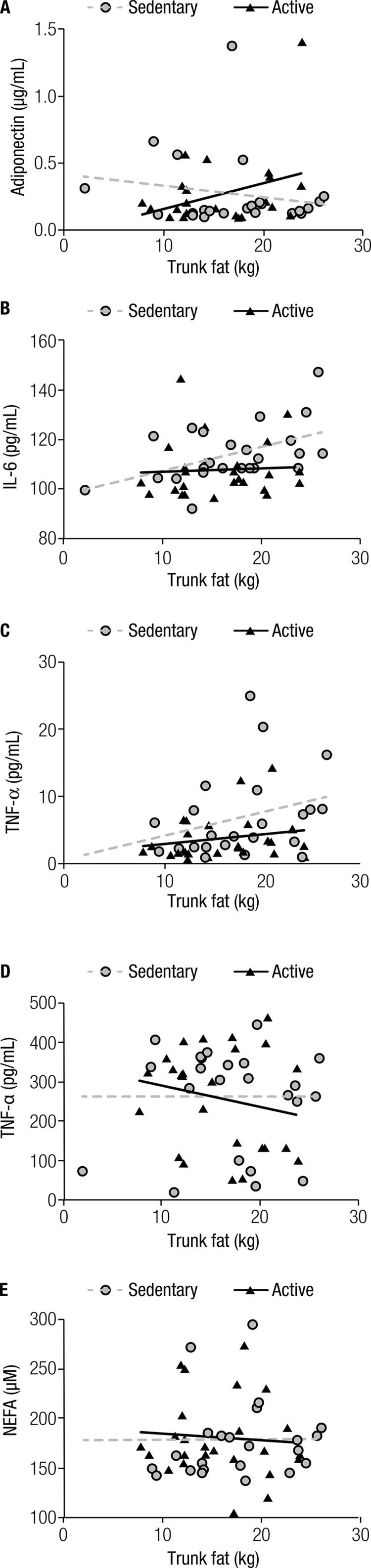
Correlations between markers of inflammation and components of body composition dichotomized by the level of physical activity. Sedentary group (n = 24) and physically active group (n = 25). (A) Trunk fat vs. adiponectin: sedentary (rho = -0.044; p = 0.837) and active (rho = 0.176; p = 0.400). (B) Trunk fat vs. IL-6: sedentary (rho = 0.471; p = 0.020) and active (rho = 0.156; p = 0.456). (C) Trunk fat vs. HOMA-IR: sedentary (rho = 0.418; p = 0.042) and active (rho = 0.198; p = 0.342). (D) Trunk fat vs. TNF-α: sedentary (rho = -0.164; p = 0.443) and active (rho = -0.099; p = 0.637). (E) Trunk fat vs. NEFA: sedentary (rho = 0.221; p = 0.300) and active (rho = -0.114; p = 0.588).

Table 2 shows the relationship between inflammation markers and central adiposity in 49 postmenopausal women. There were positive and significant correlations between trunk fat and both IL-6 and HOMA-IR. We further investigated the relationship between metabolic and inflammatory profiles and central adiposity dichotomized by MVPA level, according to ACSM recommendations ( Figure 1 ). Interestingly, a positive association between proinflammatory cytokines and trunk fat was only found in sedentary women. Moreover, physically active women presented a positive association between percentage of body fat and adiponectin (p = 0.027; data not shown).

**Table 2:** Correlations between markers of inflammation and central adiposity (n = 49)

	Trunk fat (kg)	

	rho	p-value
Adiponectin (μg/mL)	0.044	0.766
IL-6 (μg/mL)	**0.300**	**0.033**
HOMA-IR	**0.295**	**0.040**
NEFA (µM)	0.078	0.595
TNF-α (pg/mL)	-0.114	0.437

NEFA: nonesterified fatty acids; TNF-α: tumor necrosis factor-α; IL-6: interleukin-6; HOMA-IR: homeostatic model assessment-insulin resistance; HOMA-β: homeostatic model assessment-β-cell function.

**Figure 2:**
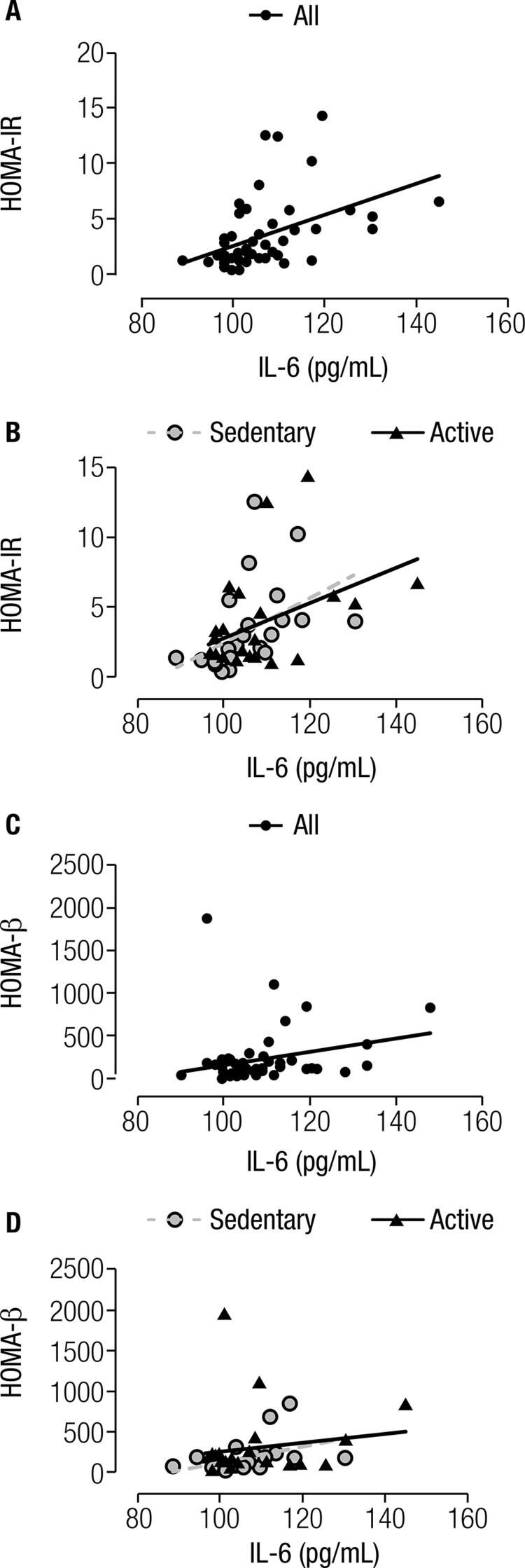
Correlations between Interleukin-6 and insulin resistance index. Sedentary group (n = 24) and physically active group (n = 25). (A) Relationship between IL-6 and HOMA-IR (rho = 0.550; p-value < 0.001). (B) Relationship between IL-6 and HOMA-IR dichotomized according to the physical activity level (sedentary: rho = 0.739; p-value < 0.001 and active: rho = 0,339; p-value = 0.098). (C) Relationship between IL-6 and HOMA-β (rho = 0.296; p-value = 0.039). (D) Relationship between IL-6 and HOMA-β dichotomized according to the physical activity level (sedentary: rho = 0.494; p-value = 0.014 and active: rho = 0.082; p-value = 0.697).

We also found a positive and moderate correlation between IL-6 and HOMA-IR (rho = 0.550; p < 0.001), and a weak correlation with HOMA-β (rho = 0.296; p = 0.039). When we performed these analyses dichotomizing the participants by MVPA level, we found higher and positive correlation values between IL-6 and HOMA-IR in sedentary women (rho = 0.739; p < 0.001), in addition to moderate and positive but not significant correlation values among active women (rho = 0.339; p = 0.098). Additionally, when we correlated IL-6 and HOMA-β according to MVPA level, we found that sedentary women presented moderate and positive correlation values (rho = 0.494; p = 0.014), whereas active women presented low correlation values (rho = 0.082; p = 0.697) ( Figure 2 ).

**Figure 3:**
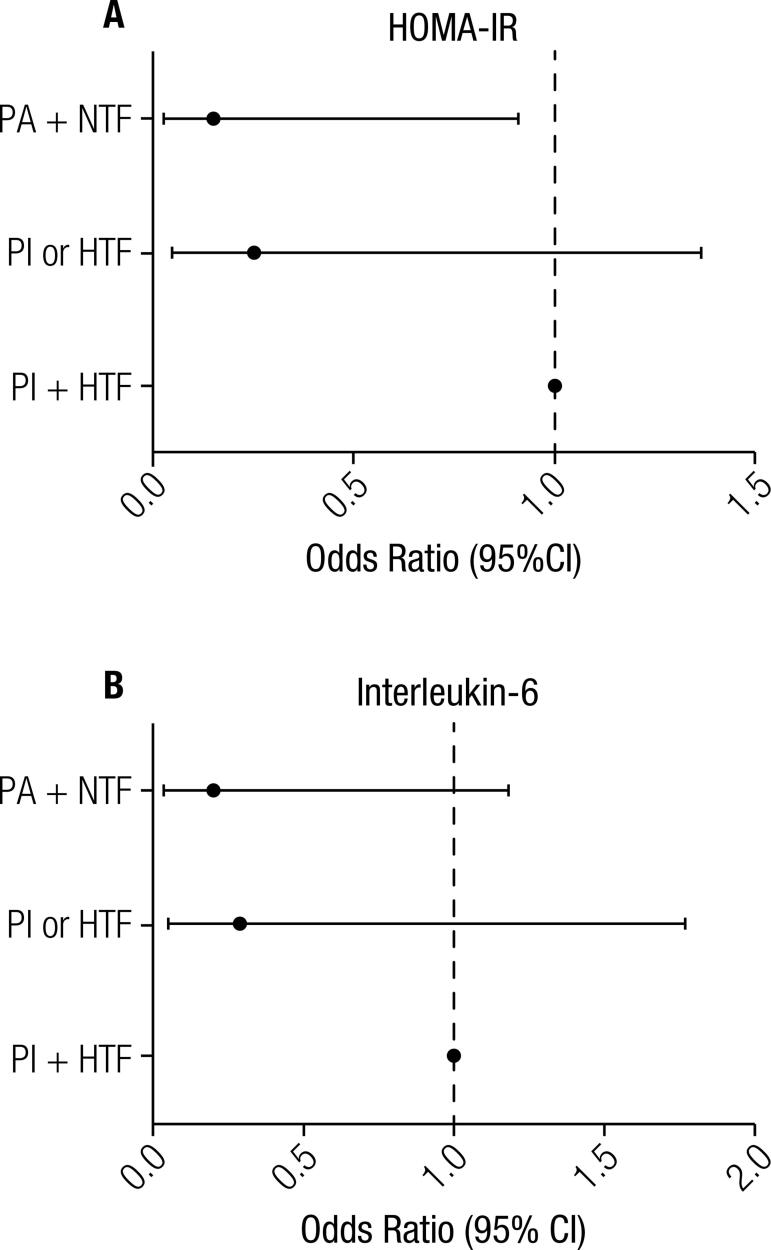
Binary logistic regression between inflammation markers, engaging in moderate-to-vigorous physical activity and trunk fat. PA: physically active women; NTF: normal trunk fat; PI: physically inactive women; HTF: higher trunk fat; 95%CI = 95% Confidence Interval.

Figure 3 shows a binary logistic regression analysis between inflammation markers, MVPA level, and trunk fat. Participants who were physically active and had normal trunk fat values presented a 14.7% lower chance of having higher HOMA-IR levels (β [95%CI] = 0.147 [0.027; 0.811]). Having no risk factors (increased trunk fat or low physical activity) did not protect against higher IL-6 levels, although this relationship presented a trend towards significance (β [95%CI] = 0.198 [0.033; 1.181]).

## DISCUSSION

The novelty of this study is the investigation of the protective factor of sufficient MVPA levels and normal trunk fat amount in the immunometabolic profile of postmenopausal women. We demonstrated that 1) the HOMA-IR and IL-6 were only associated with trunk fat in sedentary women, and 2) the protective effect of MVPA levels and a normal trunk fat amount was observed in association with the HOMA-IR but not with IL-6 concentrations.

Sedentary behavior is recognized as one of the most powerful risk factors for metabolic diseases ( 23 , 24 ). It is also known that the lack of physical activity is independently associated with a worse immunometabolic profile ( 5 , 25 , 26 ), and when this behavior is clustered with obesity, the risk of death increases 7.5 times ( 23 ). On the other hand, independently of sex and age, being moderately active results in 26% and 27% lower chance of death in overweight and obese subjects, respectively ( 23 ). In the present study, sedentary women showed a significantly positive association between trunk fat, HOMA-IR, and IL-6, which may indicate the occurrence of immunometabolic disorders. These findings are in agreement with those by Lavoie and cols *.* ( 27 ), who demonstrated that physical activity plays an important role in reducing chronic low-grade inflammation and risk of metabolic and cardiovascular diseases. Furthermore, women with more than one risk factor, including low physical activity levels, are more likely to present metabolic disorders ( 13 ).

In contrast, physically active women presented no association between trunk fat and immunometabolic profile, possibly indicating that these women were protected against immunometabolic disorders. Physically active women also presented a positive association between percentage of body fat and adiponectin, an anti-inflammatory cytokine secreted mainly by adipocytes ( 28 ). Although increased body fat attenuates the expression of adiponectin ( 9 ), some types of acute physical activity can increase adiponectin levels independently of weight ( 29 - 32 ), possibly indicating that the anti-inflammatory effect of physical activity overcomes the detrimental effects of excessive fat mass.

Analyzing odds ratios, we found that women who were physically active and had normal trunk fat values presented a protective profile against high HOMA-IR but not IL-6. We also observed that having only one factor, sufficient physical activity levels (MVPA ≥ 150 min/wk) or normal trunk fat, was not enough to carry over the protective effect on immunometabolic disorders. Our results are in line with those by Knudsen and cols *.* ( 33 ), who found that only 3 days of reduction in physical activity levels led to increased insulin concentration and decreased insulin sensitivity. Interestingly, this pattern occurred regardless of the increase in body fat (visceral and abdominal), indicating the powerful harm of sedentary behavior. In contrast, the authors observed no changes in IL-6 levels in these patients. As already demonstrated, a drastic reduction in physical activity can attenuate the peripheral insulin sensitivity by decreasing the phosphorylation and content of Akt, an important downstream protein in insulin signaling and glucose uptake ( 34 ). However, the same authors have also found no relationship between a reduction in physical activity and IL-6 ( 34 ). The absence of an association between IL-6 and physical activity in these studies might have been due to the fact that a drastic reduction in physical activity was not enough to increase fat mass, which is one of the factors responsible for the IL-6 increase ( 24 ).

Despite the significance of our findings, it is important to mention some limitations of our study. The sample size indicates a need for caution in extrapolating the results, and the cross-sectional design precludes longitudinal considerations and causal inferences. On the other hand, the positive aspects of this study should be highlighted. Perhaps the most notable was the objective measurement of physical activity using triaxial accelerometer, providing a reliable measurement of habitual physical activity and avoiding eventual mistakes presented in subjective self-reported physical activity questionnaires. Furthermore, the estimation of body composition by DXA is a strength of the study, since this is a reliable and highly precise method to assess the body composition of the studied population ( 35 ).

In summary, our results suggest that sufficient MVPA levels were a protective factor against immunometabolic disorders in postmenopausal women. Therefore, steps must be taken to stimulate the practice of regular MVPA, especially in this population.
